# ‘Geophagy’ and Clay Minerals: Influencing Ruminal Microbial Fermentation for Methane Mitigation

**DOI:** 10.3390/microorganisms13040866

**Published:** 2025-04-10

**Authors:** Zubaer Hosen, Md. Rashidul Islam, Ravi Naidu, Bhabananda Biswas

**Affiliations:** 1 Global Centre for Environmental Remediation, School of Environmental and Life Sciences, The University of Newcastle, Callaghan, NSW 2308, Australia; mdzubaer.hosen@uon.edu.au (Z.H.); mdrashidul.islam@newcastle.edu.au (M.R.I.); ravi.naidu@newcastle.edu.au (R.N.); 2 crc for Contamination Assessment and Remediation of the Environment (crcCARE), University Drive, Callaghan, NSW 2308, Australia

**Keywords:** rumen, methane, geophagy, clay minerals, microbes, clay–microbes

## Abstract

Methane is a greenhouse gas with high warming potential, and ruminants like cattle and sheep are a major source of its emission. In the rumen, the first stomach compartment, diverse microorganisms and fauna live, including archaea, bacteria, protozoa, nematodes, and fungi. They participate in complex fermentation processes. During rumen fermentation, various gases are produced, dominantly hydrogen and carbon dioxide. In methanogenesis, methanogens utilize these two gases to produce methane as a byproduct, which burps out into the atmosphere. Therefore, interfering with this methanogenesis is a promising way of reducing methane. Supplementing feed containing clay minerals could be one of method to do so as ruminants naturally consume them as they graze, often called “geophagy”. This review discusses the role of clay minerals in enteric methane abatement, emphasizing the clay–microbial interaction in the rumen. In these interactions, clay minerals also serve as a carrier for other chemicals and influence microbial attachment. Elemental dissolution and cations from clay mineral and their buffering capacity can further influence microbial dynamics in rumen fluids. By combining insights from microbiology, soil science, and animal nutrition, this review provides an interdisciplinary view of rumen interactions. Findings from this review can help to develop a low-cost and safe clay feed supplement to reduce livestock methane emissions.

## 1. Introduction

Methane (CH_4_) is a greenhouse gas with a significantly higher global warming potential (GWP) than carbon dioxide (CO_2_) over a 20-year horizon, trapping approximately 85 times more heat than an equivalent amount of CO_2_ [1,2]. Various sources, such as livestock production, industry, and mining, have largely contributed to its atmospheric emission. This emission has risen significantly, by approximately 20% in recent decades, primarily due to the expansion of these major industry sectors [3,4,5]. Among them, the agriculture sector contributes to approximately 40% of total anthropogenic CH_4_ emissions, mostly through “burping or belching” as a result of enteric fermentation in ruminants such as cattle, sheep, and goats. The CH_4_ emission reduction in livestock is crucial for environmental sustainability. However, considering the significance of the livestock industry in food sources and other agribusiness, the downgrade in the livestock production is not an option. This CH_4_ burping as a result of fermentation is mostly microbe-mediated, where CH_4_-producing microorganisms are involved. The process is known as methanogenesis, where other physicochemical and dietary factors such as pH, oxidation reduction potential (ORP), electrical conductivity (EC), dietary fiber, supplements, fat, and protein content in the feed also influence the rate and amount of CH_4_ generated [6].

These factors can be controlled by developing an appropriate mitigation approach. The current research and development outcomes favorably suggest that intervening in the “methanogenesis” process would be an “feasible” option for curbing enteric CH_4_ burps. Several potential mitigation strategies have often been confined to dietary ingredients and supplements. These include the use of chemical inhibitors, microbial inoculum, alternative feed sources, and mineral additives that can reduce methanogenesis [7]. Among chemical inhibitors, 3-NOP and bromoform appear to be in the advanced stages of research but show potential toxicity, as well as difficulties in mass production and on-farm adaptation [8,9,10]. A few groups of microbes, including sulfate-reducing bacteria, acetogen, propionate-forming bacteria, and nitrate-/nitrite-reducing bacteria have also been tested for possible ruminal intervention [11]. These biochemical interventions for CH_4_ suppression can also be achieved using feed additives, such as clay minerals (CMs), plant extracts, and dietary chemicals [7,10].

Among these, all the dietary additives are outside of the scope of this review except for CMs. The CMs are an integral part of soils, composed of aluminosilicate layer, which are low cost and non-toxic to animals. The motivation for this review came from a historical behavior of animals, a practice known as ‘geophagy’, where a significant amount of surface soils or CMs are advertently or inadvertently consumed by grazing animals. Consequently, increased number of research attempts have been made where various raw and activated or processed CMs were utilized as feed additives to reduce livestock CH_4_ emissions [12,13,14,15]. Further, CMs are not only proven for ruminal CH_4_ suppression but also demonstrated for livestock health promotion by regulating the activities of gut microbes as well as enteric pathogens [16,17,18,19]. Therefore, delicate applications of CMs in enteric CH_4_ inhibition require a critical understanding of the reactivity and fate of these materials in live ruminants [18,19,20,21,22]. Despite literature documenting this application of CMs, the scientific community is trying to understand how the CMs interact with the methanogenesis process, considering the extremely complex microenvironment in the rumen and other digestive tracts of ruminant animals. Therefore, this review will consolidate these interactions as well as discuss possible mechanisms from interdisciplinary points of view, such as known physicochemical properties of CMs and the growth and functions of ruminal microorganisms.

## 2. Biological Processes of Ruminal Methane Production

**Figure 1:**
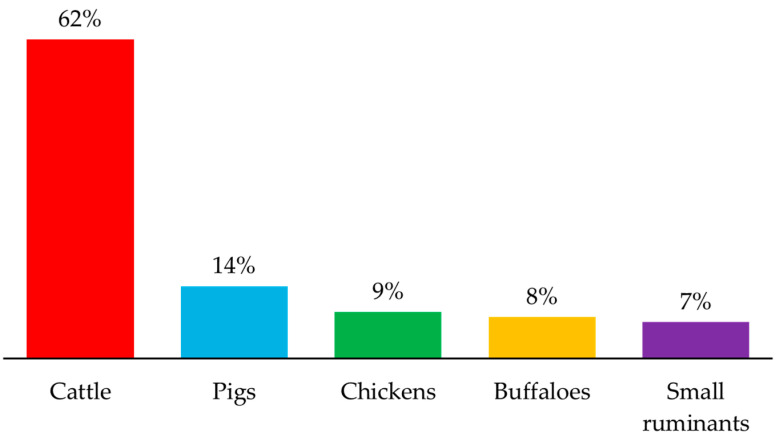
Livestock contributions to CH_4_ emissions. Graph has been generated from data reported by FAO [24].

The emissions of CH_4_ gas from ruminant sources (e.g., dairy and non-dairy cattle, buffalo, sheep, and goats) are a major concern. This emission represents 39% of the total GHG from the livestock sector [7,23]. Amongst livestock, cattle are a large contributor to CH_4_ emissions compared to other animals (Figure 1). Due to the rising global demand for meat, milk, and other agri-services, the ruminant industry has increased animal production, resulting in over two-thirds of total CH_4_ emissions from all livestock animals [24].

**Figure 2:**
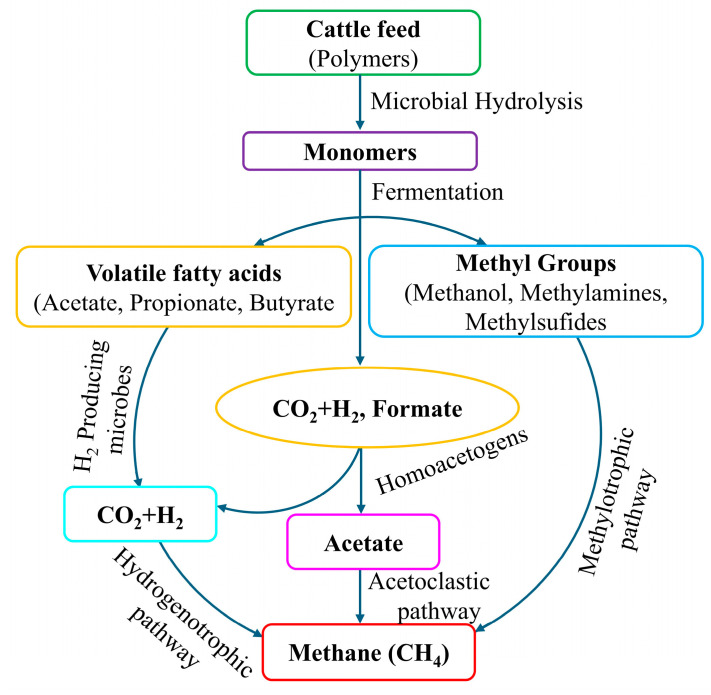
Production of CH_4_ through anaerobic fermentation of biopolymer, which has been adopted and modified from Kumar and Paswan [31].

Rumen methanogenesis is influenced by a group of anaerobic microorganisms, including methanogenic archaea, bacteria, protozoa, nematodes, and fungi. Among them, archaea mostly contribute to the methanogenesis process, whereas bacteria, protozoa, nematodes, and fungi also variably participate in this process [25,26]. In this diverse context of methanogens, scientists reported that the rumen has a dominant characteristic of archaea, accounting for over 60% of total [27,28]. *Methanobrevibacter ruminantium* and *Methanomicrobium mobile* are two such dominant archaea in rumen environment [29,30]. Figure 2 shows that rumen methanogens utilize predominantly hydrogen (H_2_) and CO_2_ as substrates after rumen fermentation to produce CH_4_ [31]. This mechanism is referred to as the hydrogenotrophic pathway [32]. Some other types of methanogenesis also occur in the rumen. These include methylotrophic and acetoclastic pathways [31].

In ruminants, bloating during the belching of grass is the primary route of CH_4_ emission to the atmosphere [33,34], thanks to the normal physiology of the ruminal digestion. Therefore, the reduction of CH_4_ generation in the rumen without impacting normal digestion or belching could be an important consideration. Higher activities of the methanogens lead to increased CH_4_ production [35], and when the methanogens are inhibited, the production of CH_4_ slows down, which may leave an excess amount of H_2_ in the rumen system [36]. This H_2_ may compensate for the generation of volatile fatty acids (VFAs), which are also considered significant H_2_ sinkers to reduce enteric CH_4_ production [37,38].

These biological processes are complex. However, a few important factors associated with these have been studied, especially to understand potential methane abatement strategies. Diet composition, fiber content, passage rate, ruminal pH, and seasonal variation are a few of them. Microbial populations influenced by diet composition can be directly linked to methane generation [39,40]. And high-fiber diets, such as those with high levels of roughage or forage, usually lead to increased CH_4_ emissions due to a shift in microbial fermentation towards CH_4_-producing archaea. In this case, high-fiber diets promote the growth of some specific microbes, such as cellulolytic (fiber-degrading) bacteria, protozoa, and fungi, which are responsible for breaking down fibrous plant material such as cellulose and hemicellulose in the rumen [41]. These cellulolytic microbes also play a key role in fermentation or rumen digestion, and their fermentative byproducts, such as H_2_ and CO_2_, can be converted into CH_4_ by the activity of methanogens. In contrast, concentrate diets, such as those of grains, promote shifting bacterial communities toward starch-fermenting species. This change increases propionate and butyrate production through succinate–CoA synthetase and pyruvate–ferredoxin oxidoreductase [37]. These increased VFAs lower rumen pH (<6.0), which makes an acidic rumen environment (acidosis) that further alters the microbial composition and reduces CH_4_ emission [42,43]. Studies show that forage/fiber-based diets can contribute up to 30% higher CH_4_ emissions than grain-based diets [44]. Nevertheless, methanogens and other microorganisms depend on each other in a complex rumen ecosystem, often termed as “symbiotic relationship” [45], which can contribute up to 37% of rumen CH_4_ emissions [46]. For example, Protozoa generate H_2_ during carbohydrate fermentation via hydrogenosomes, which methanogens (*Methanobrevibacter* spp.) utilize for their metabolic requirements [25,47], and generate around 15–35% CH_4_ [48]. Cellulolytic bacteria, such as *Ruminococcus* and *Fibrobacter* spp., degrade cellulose, releasing H_2_ and CO_2_ for methanogens [47]. Moreover, a slow passage rate of feed allows prolonged interaction between feed particles and the rumen microbiota, promoting methanogenesis [49]. In contrast, a faster passage rate reduces this microbial interaction time, limiting CH_4_ formation. Controlling H_2_ utilization by methanogens can lead to inhibit the methanogenesis process.

Furthermore, ruminal pH plays a key role in maintaining a balanced microbial population and fermentation processes, which is directly linked to CH_4_ production. Although pH in the rumen environment depends on the type of diet, pH ~5.5–7.0 is often considered an ideal pH [50], and the methanogens are optimally active between pH 6.0 and 8.0 [51]. When the pH is <6.0, the production of CH_4_ decreases, and it starts increasing with the increase of pH [52]. Environmental factors such as summer and winter also significantly influence enteric CH_4_ production in the ruminants, mainly as a result of temperature fluctuations. High temperature usually during summer increases the hunger of the cattle through the secretion of the ghrelin (hunger) hormone [53], and intake of higher percentages of grass or forage diet from the pasture. Whether controlling ruminal microbes, pH, and passage time of the other anti-methanogenic ingredients, intake of CMs by the ruminant animals has benefits for both methane abatement as well as rumen health.

## 3. Use of Clays in the Enteric Methane Abatement

### 3.1. Overview of the Properties of Clay Mineral

**Figure 3:**
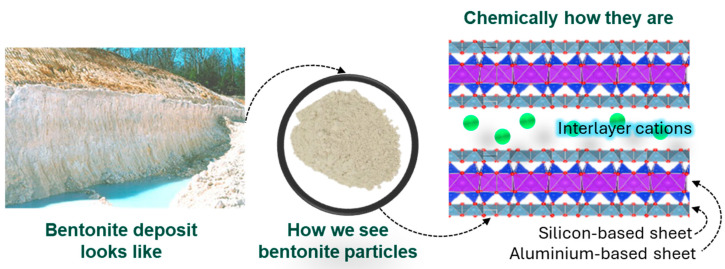
Basic physical and chemical structure of clay minerals. Bentonite clay is used as an example to showcase properties.

Soils, sediments, and rocks on Earth’s surface and in its brittle upper crust contain a wealth of clay minerals [54]. These fine-grained hydrous phyllosilicates are naturally occurring; they are plastic when mixed with water and harden when dried or fired. The basic building blocks of CM are sheet-like layers consist of silica tetrahedral sheets and aluminum or magnesium octahedral sheets [55] (Figure 3). These layers are formed by sharing oxygen atoms between adjacent tetrahedral and octahedral sheets. These sheets are attached with van der Waals and ionic integrations. Depending on the chemical composition and structure of the CMs, the most common structures are 1:1 and 2:1. In 1:1 CM, one tetrahedral sheet is attached to another octahedral sheet. The best example of this CM structure is kaolinite (Table 1). On the other hand, one octahedral sheet is sandwiched between two tetrahedral sheets in 2:1 type CMs; illite, smectite, and saponite are examples of this kind [55] (Table 1). Moreover, depending on the electric charge of the sheet and pH medium, CMs can be grouped into three groups as a result of their net either positive or negative and neutrally charge [56]. For instance, smectite typically carries a net negative charge, whereas layered double hydroxides (LDH) exhibit an overall positive charge, and talc or specific types of kaolinite may present as neutral under particular pH levels (Table 1).

Furthermore, certain CMs are rich in specific elements and ions. For example, smectite, often commercially known as bentonite, can be Na-rich (with montmorillonite as the main constituent) or Mg-rich, containing saponite. Similarly, kaolin clay primarily consists of kaolinite and nanotubular halloysite (HNTs). In all cases, impurities such as iron-bearing compounds may be present [58]. Morphologically, they also exhibit distinct properties. For example, kaolinite has a platy aluminosilicate structure, while HNTs have hollow tubes with a negatively charged siloxane surface on the outside and a positively charged aluminol surface inside the lumen [59]. In terms of utilization of these minerals, nearly 30 categories of clay deposits are available worldwide, classified by mineral composition, particle size, plasticity, color, and other properties that suit a wide range of industrial, agricultural, and artistic applications. The primary categories include both natural and purified forms [60]. For use, clay minerals are often used as particles, such as in the form of powder or colloidal solution (Figure 3).

### 3.2. Clay Minerals into “Geophagy” and Ruminal CH_4_ Mitigation

Geophagy is a natural habit of animals consuming soils and/or CMs. All herbivorous animals, including ruminants, inadvertently ingest a significant amount of topsoil without facing any side effects [12]. Moreover, animals often deliberately consume top soils or minerals to reduce their diarrhea and uncomfortable situations in their gut [7]. Recent studies showed that CMs can suppress ruminal CH_4_ emission as well [18,61]. However, the amount of clay consumed during grazing may not be sufficient to effectively reduce this enteric CH_4_ emission. In addition, the efficacy of the raw clay, which is consumed during grazing, is very poor, which encourages researchers to provide it as a feed supplement in raw or modified forms. Natural kaolin clay has greater acceptance than other clays because of its high availability, low cost, rapid ion exchange activity, unique nanosheet morphology, predominance in geophagy, and higher palatability [12,62]. Furthermore, it can serve as an anti-toxin agent, anti-diarrheal supplement, and adsorb trace metals while maintaining a neutral pH [63]. Raw CMs and their modified forms such as that are obtained chemically, mechanically, or both can adsorb a wide variety of substances due to their specific structural and surface physicochemical properties. For these reasons, these minerals are of research interest for developing a safe supplement in the methane abatement program [64].

### 3.3. Supplementation of the Clay Minerals as Specialized Cattle Feed

Clay minerals contain various essential elements or ions, such as Fe, Na, Ca, Mg, and K, and their forms which can serve as nutrient sources for animals. The inclusion of CMs in ruminant diets can also significantly influence ruminal microbial populations and their metabolic activities. Specific clays and related minerals, such as kaolinite, saponite, bentonite, and zeolite, have been studied for their effects on ruminal fermentation parameters and microbial dynamics. Cattle showed reduced CH_4_ emission in their burps when their basal feed contained raw CMs. Similar results were also obtained when other dietary additives that contained CMs were provided as a mix of basal diets. In both cases, this enteric CH_4_ inhibition was achieved even when the high forage diet was fed [12,22,65,66]. In this context, clay can shift microbial fermentation pathways toward propionate rather than acetate. Propionate formation does not produce hydrogen, unlike acetate, which is a major hydrogen generator in the rumen. By supporting propionate production, clay supplementation lowers hydrogen availability, thereby decreasing CH_4_ formation [7]. Consequently, Pikhtirova et al. [13] reported that adding 0.15 and 0.25 g of saponite clay to the fermentation mixture has been associated with reduced CH_4_ production without altering total volatile fatty acid (VFA) concentrations, suggesting a shift in microbial fermentation pathways. The reduction in methane emissions may be linked to decreased activity of methanogenic archaea, such as *Methanobrevibacter ruminantium* and *Methanosarcina barkeri*, which utilize hydrogen to produce methane [67].

Clays like bentonite show high adsorption and cation-exchange capacity, allowing them to effectively adsorb ammonia and other cations. This adsorption can modulate the availability of ammonia in the rumen, potentially affecting the growth of ammonia-utilizing bacteria. Studies have shown that bentonite supplementation can lead to a reduction in ammonia concentrations during fermentation, which may influence the proliferation of proteolytic bacteria such as *Butyrivibrio fibrisolvens* [22]. In addition, clays, due to their buffering capacity, can suppress the growth and activity of symbiotic organisms like protozoa, which indirectly contributes to rumen CH_4_ emission, while preserving beneficial microbes that support healthy rumen fermentation [13,15,22,68]. In this case, inclusion of clay such as bentonite and zeolite in the ruminant diet has been associated with stable pH levels, especially during the initial stages of fermentation. This buffering effect creates a more suitable environment for cellulolytic bacteria, such as *Fibrobacter succinogenes* and *Ruminococcus albus*, which are sensitive to low pH conditions. By maintaining an optimal pH, both clays support fiber degradation and overall fermentation efficiency [22]. Moreover, a laboratory-based experiment demonstrated that both pristine kaolinite and iron-coated kaolinite exhibited inhibitory effects on microbial methanogenesis, specifically targeting *Methanosarcina mazei* and *Methanothermobacter thermautotrophicus* [69]. These studies indicate that while clay supplementation can influence the rumen microbial ecosystem and fermentation parameters, limited specific information exists on its effects on methanogenic archaea and associated gene expression.

**Figure 4:**
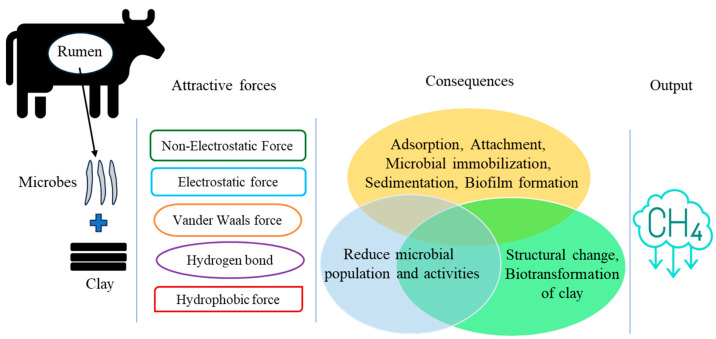
Schematic representation of potential clay–microbe interactions to reduce CH_4_ production.

The use of CMs in the cattle diet is not an entirely new approach in conventional and intensive growing practices. However, scientists have been trying to make these CMs for additional or “specialized” supplements, aiming for them to be CH_4_ inhibitory. Compared to other soil minerals, CMs possess a higher specific surface area, CEC, and surface charge density [70]. These properties are pivotal for target applications, including animal husbandry and CH_4_ reduction [7,16,17,18,19]. The net surface charge of CMs is usually negative, which may adsorb positive ions like hydrogen (H^+^). From the mechanistic point of view, clays may interact with microbial cell membranes through van der Waals forces, electrostatic or hydrophobic interactions, and hydrogen bonding in the rumen environment [19] (Figure 4). However, the insight mechanism is very complex for the rumen environment and not yet clearly reported. Moreover, the specific properties of clays that reduce ruminal CH_4_ production, and the very delineate clay-based CH_4_ reduction mechanism, are also unclear.

### 3.4. Raw and Modified Clay Minerals to Carry and Deliver Other CH_4_ Inhibitory Additives

The use of chemical feed additives, such as ionophores, 3-nitrooxypropanol (3-NOP), and nitrogenated and halogenated compounds, has also been reported to inhibit CH_4_ production in the rumen by altering microbial populations and metabolic pathways [7,10]. The ionophores reduce bloating, acidosis [71], H_2_ availability (nutrients for methanogens) [72], and ciliate protozoal population, which hinder the activities of methanogens and thereby decrease CH_4_ generation [73]. In an anaerobic methanogenesis pathway, the catalytic activities of methyl-coenzyme M reductase (McR) include numerous reactions through nickel and other coenzymes, releasing CH_4_ in the rumen [74]. Inhibiting any steps of this reaction can disrupt the methanogenesis pathways. Supplementation of 3-NOP in the ruminants’ diet inhibits the normal maturation of methanogens without influencing the non-methanogens. In this case, 3-NOP can bind with the active site of the McR and alter the normal methanogenic pathway. Therefore, it gradually inactivates McR [75], which results in the reduction of enteric CH_4_ production. During this process, microbes responsible for the digestion of feedstuffs can tolerate nitro toxin, and they can maintain their growth and activities in the rumen [76]. Enteric CH_4_ emissions were decreased by 20–60% variably based on the applied methods and duration of the dosage. Moreover, other types of nitro compounds with similar characteristics, such as nitroethane, and 3-nitropropionic acid, have been experimented with as methanogen inhibitors [77]. Furthermore, one in vitro study demonstrated that halogenated sulfonate compounds (e.g., bromoethane sulfonate and bromopropanesulfonic acid) reduced nearly 80% of CH_4_ emissions without impacting the ruminal digestion [71,78]. Beyond them, tannins, saponins, oils, and plant extractants were also studied for the ruminal CH_4_ reduction. However, their long-term use is often not recommended due to concerns about animal health, nutrient utilization, variability in effectiveness, potential toxicity, cost, and regulatory issues [79,80].

Clay minerals are widely known as potential safe materials to deliver other ingredients or chemicals [19,81,82]. Considering this, they can also be used as the carrier or co-binder of those methane-inhibitory chemicals [83]. By delivering CH_4_-inhibitory substances, such as plant extracts, specific essential oils, or other chemical inhibitors, as mentioned above, CMs can help reduce CH_4_ production by interacting directly with methanogens or altering rumen microbial populations to favor less CH_4_-intensive pathways. However, modification of natural clays using mechanical, chemical, or biochemical processes enhances their physicochemical properties, making them more effective in various applications [16,61,81,84,85,86]. Additionally, modifying or intercalating CMs with CH_4_ inhibitors may enable the slow release of these compounds, minimizing the potential adverse effects of high-dose, fast-acting additives on animal health [84,85,86,87]. This approach not only promotes safer administration but also enhances CH_4_ suppression through synergistic effects. However, future studies are encouraged to further explore how different clay structures and particle sizes interact with microbial communities in the rumen to optimize CH_4_ inhibition without adverse effects on ruminant health and performance.

### 3.5. Stakeholders’ Acceptability of Clay-Based Cattle Diet

Clay and zeolite are commonly used in the livestock feed industry. They are added as co-ingredients in lick blocks and loose licks, primarily for urea, phosphorus, and other micronutrient supplements [88]. Clays, with their buffering capacity, can reduce acidosis, while holding and delivering those nutrients [13,15,68,89,90]. Therefore, using a modified form of clay minerals as a methane inhibitor is not a significant deviation from on-farm adaptation and stakeholder acceptability. However, clays produced via aggressive chemical methods require a safety evaluation before being added to lick blocks or loose licks.

Regardless of whether CMs are used as raw materials or other ingredient carriers for methane abatement supplements, their inclusion in the animal digestive system involves known, simulative, and potentially many unknown interactions with ruminal and other digestive tract microbiota. These interactions may not be exclusive to methanogenic microorganisms but are useful for understanding the fate of clay minerals and potentially increasing the acceptability of clay-based methane-inhibitory diets.

## 4. Clay Minerals and Their Interactions with Rumen Microbiota

### 4.1. Possible Mechanisms of Clay–Microbe Interactions in the Rumen

Surface properties of clays, such as CEC, specific surface area, adsorption capacity, electron transfer media, solution buffering properties, and binding capacity for ions, which can facilitate the clay–microbial interaction process in the rumen [7,15,19]. These interactions can be performed by electrostatic or non-electrostatic attraction and attachment to the clay surface (Figure 4). These attachments occur through cation bridges, facilitated by the release of interlayer metal cations from CMs, which may increase EC [13,61]. Moreover, microbial interaction with clays can also take place through hydrophobic interactions, ion exchange, and electron transfer can be the predominant driving forces [91]. The consequences of clay–microbe interactions, such as microbial attachment, dissolution, sedimentation, biotransformation, mobilization, and immobilization, have been widely reported for environmental matrix (e.g., soil and/or water) in the presence of both aerobic and anaerobic conditions, including the insight mechanisms [91,92].

**Figure 5:**
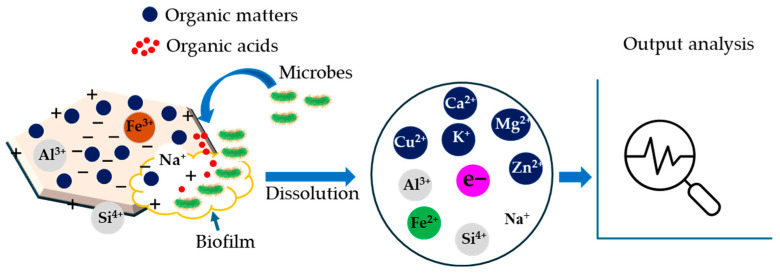
Possible dissolution mechanism through clay–microbe interactions in the rumen. Theme of this scheme was adopted from published journals [69,97].

These mechanisms can often be applied in other aqueous environments like the rumen as an anaerobic fermentation media. However, the specific mechanisms and extent of dissolution can vary depending on the type of clay, the conditions within the rumen (such as pH and microbial activity), and the diet of the animal [93]. Moreover, microorganisms can cause structural changes in the smectite through mechanisms like biomineralization, reductive dissolution, and sorption processes (Figure 5). These interactions alter the crystal structure, which can be difficult to quantify without advanced tools. For example, highly reactive metal oxides, especially iron (III) in the ferric salt, can instigate iron-reducing bacteria that compete with methanogens due to the higher affinity of iron-reducing bacteria to their common substrates such as acetate and hydrogen [69]. Reports demonstrated that methanogens could reduce structural Fe (III) in iron-bearing smectite, resulting in the alteration of their CH_4_ production [94,95,96].

In the ruminal digestion process, some alumina silicate minerals dissolved in the ruminal fluid and released trivalent Al^3+^ ions, which are considered a methanogenesis inhibitor [98]. The phyllosilicate edges were observed to be associated to a greater extent with the predominant attachment of the cells and/or with facilitated mineral dissolution compared to the basal planes [99]. Moreover, raw and modified clays can be an effective rumen modifier that have shown different interactions with rumen microbes and parasites (Table 2). These interactions include microbial adhesions and dissolution of CMs [19]. After adhesion or attachment onto the clay surface, the microbe secretes extracellular polymeric substance (EPS) and forms colonization [100]. On the other hand, by hydrophobic interactions, the EPS layer and protein-binding receptors, EPS highly influence CMs due to its slimy texture and ionic charges when they come closer [97,101]. Sometimes, the primary elements of clay minerals (e.g., Al and Si) also take part in the bond formation with the functional group of microbial cells. In the rumen environment, the interaction between clay and microbes is ambiguous, and different controversial statements have been reported in both in vitro and in vivo experiments. For example, some studies indicated that supplementation of bentonite, zeolite, saponite, and sepiolite increases ruminal performances such as rumen fermentation, digestibility, metabolism, and ruminal production (e.g., meat and milk) across various dietary ratios [13,17,22,102,103,104], though other studies have shown some negative impacts on nutrient digestibility in the rumen, particularly when using high dosages [61].

The ions (either cations or anions) present in the CMs can interact with microbes to exert a negative or positive impact on a particular species. For example, two prevalent mechanisms of ferric iron reduction from smectite clays under microbial interaction, such as solid-state reduction and dissolution–precipitation have been observed [109,110]. After the formation of a stable biofilm, the ferric iron coating on the clay surface is released as a soluble ferrous form by the activities of slow-released organic acids from the microbes (Figure 5). This process is called the dissolution of CMs [69]. However, the dissolution mechanism of raw and modified clays has not been clearly documented in the literature for a ruminal environment (Table 2).

Considering the complex biochemical nature of rumens, it is assumed that CMs can influence the movement of essential nutrients for the ruminant microbes [111], while the nutritional requirement for methanogenic microbes may be different from other microbes. The clay particles in the digestive system can play a role in competition, attraction, or repulsion phenomena. For instance, organic acids act as a ligand to release nutrients, and CMs can intervene in both the adsorption and leaching process [91,112]. This regulates the nutrients available to microorganisms in the anaerobic environment, including the rumen [99]. After taking the nutrients, the stable microbial colonies formed on the clay surface release EPS, which forms biofilm, protecting the microbes from environmental stresses and enabling their survival and growth [113,114] (Figure 5).

Types of clay minerals are likely one of the “factors of interest” in assessing the microbial profile and ruminal ecosystem in clay-amended basal diets [19,20,22,106]. Some of the effects of specific clay and clay minerals on the ruminal system have been revealed recently (Table 2). Microorganisms may find the morphology, elemental dissolution, or pH buffering effects of a particular clay mineral either beneficial or inhibitory. For example, adding a trace of CMs such as bentonite, saponite, and sepiolite to the ruminant diet provides an improved, stable, and healthy digestive system [13,22]. Although these findings were obtained from a rumen fluid simulation (in vitro) system, those CMs had a clear influence on bacterial activity compared to their controls. This effect was likely due to their pH-buffering capacity and impact on ammonia concentration. Here, bentonite (10 mg/g of total substrate) helps maintain a balanced pH in a sheep rumen but can also influence ammonia concentration, while sepiolite (10 mg/g of total substrate) supplementation may suppress microbial fermentation in high-concentrate diets, potentially reducing VFA production and gas emissions. This contrasting effect was likely due to the lower buffering capacity resulting from the presence of sepiolite compared to that from bentonite [22]. In another study using cow rumen fluid, supplementing saponite clay (0.15 and 0.25 g) did not affect ruminal pH or VFA production but significantly reduced CH_4_ [13].

Although clay-based ruminant diets are less studied, recent advances in high-throughput sequencing and metabolomics provide insights into microbial adhesion to clay particles, metabolic activity, and community dynamics. For example, in high-concentrate diets, a bentonite-supplemented diet shifts the particle-associated microbiota, maintaining microbial diversity closer to roughage-diet profiles [13,106]. In this case, adding bentonite to the basal diet reduced bacteria that are associated with low pH, such as *Lactobacillus*, while promoting the growth of beneficial groups, including *Campylobacter* and *Butyrivibrio*. This study also demonstrated bentonite’s efficacy in reducing potentially harmful bacteria, particularly Gram-negative genera such as *Treponema*, *Fusobacteria*, and *Succiniclasticum* [106]. With the growing use of clay minerals as feed supplements, studying microbial changes in the presence of specific clay species is necessary. This is largely due to the unique properties of clays, the complexity of the rumen ecosystem, and the associated analytical challenges.

### 4.2. Analytical Challenges in Studying Clay–Microbe Interactions in the Rumen

The use of natural and modified CMs as dietary supplements and their CH_4_ inhibitory functions are often judged based on the desired outcomes, such as low CH_4_ burps and no adverse effect on live animals. Additionally, the hypothesis and known properties of CMs can be explained to only some extent without unveiling details of them. This challenge lies in the analytical difficulties, thanks to the complexity of the composition of the rumen fluid and microbial ecosystem in the rumen. However, there are tools that have been used in many complex environmental samples and in the case of rumen fluids that can be applied as well. In the previous section, we discussed the mechanisms of clay–microbe interaction in detail (see Section 4.1). Now, we will focus on the tools and considerations involved in studying these interactions.

#### 4.2.1. Challenge in the Sample Collection and Preparation

Sample preparation in rumen experiments is a complex process that can impact the accuracy and reliability of microbial analysis and imaging results. Rumen samples must be kept under strict anaerobic conditions from collection through processing, as exposure to oxygen can significantly alter the microbial community composition, especially for obligate anaerobes like methanogens [115]. Specialized equipment, such as anaerobic chambers or gas-tight containers, is required to maintain these conditions, adding complexity to sample preparation. Moreover, during sampling, there is a risk of contamination from external sources or cross-contamination between samples. This can be particularly problematic when working with rumen contents, which have a complex microbial environment. Proper sterilization protocols and handling procedures are essential but can be challenging to maintain consistently [116]. Furthermore, the heterogeneous nature of rumen contents, including clay, fibrous plant material, liquids, and microbial biofilms, makes it difficult to obtain uniform samples. After introducing trace amounts of clay into the rumen solution, random sampling methods, such as scooping, are likely to yield inconsistent results [117]. This approach may not capture areas where clay and microbes are attached, making it difficult to observe or quantify these interactions accurately.

#### 4.2.2. Microscopic Imaging of Microbial Attachment on the Clay Surface

**Figure 6:**
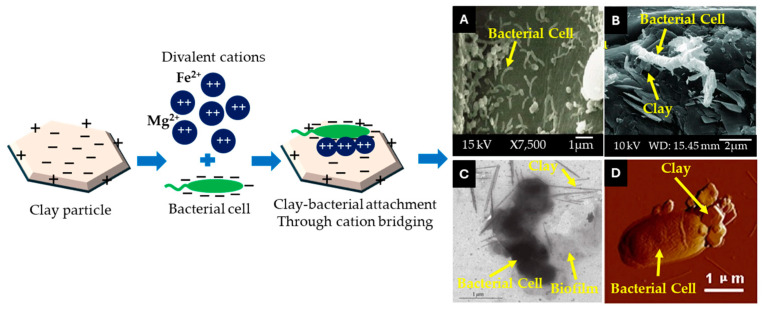
Possible ways to perceive clay–microbe attachment. (**A**–**D**) are performed by SEM, TEM, and AFM techniques, respectively. These are representative images from existing reports that have been conducted on rumen, soil, and aquatic environments [118,119,120,121,122].

Identifying which microbes attached to the clay surface and which avoided it poses arguably the most direct evidence for the immobilization of microbial cells with the clay particles and possibly the travel of the duo in the digestive system. Microscopy imaging by the use of scanning electron microscopy (SEM), transmission electron microscopy (TEM), or atomic force microscopy is needed to do so (Figure 6).

However, imaging microbes on mineral surfaces, especially in the context of rumen fluid, presents several challenges primarily due to sample preparation techniques and the inherent complexity of the fluid itself [123]. The ruminants consume a variety of plant-based diets that contain numerous pigments, which are released into the rumen fluid through different enzymatic and microbial activities. The most common pigments found in the rumen include chlorophyll, carotenoids, bilirubin, biliverdin, and urobilins, which turn the rumen fluid cloudy [124]. These pigments also obscure details or alter the electron density of the sample, impacting the visibility of microbial structures [125]. Additionally, the rheological properties of rumen fluid can pose challenges in imaging. The viscosity and particulate nature of rumen contents can hinder the uniform distribution of microbes on mineral surfaces, leading to inconsistent imaging results [116,126]. To tackle these issues, advanced imaging techniques and meticulous sample preparation methods must be employed, ensuring minimal disturbance to the microbial community while maximizing the clarity of the images obtained. This involves optimizing conditions for sample fixation, drying, and mounting to improve the quality of microbe–mineral interactions in imaging studies [125]. However, imaging techniques only reveal the microbial community interacting with CMs, leaving the non-interacting microbial community in the rumen unexplored. Therefore, combining imaging with molecular analyses, such as DNA extraction and sequencing, is essential for a comprehensive understanding of the entire scenario.

### 4.3. Fate Analysis of Ingested Clay Minerals in the Rumen or in Feces

The fate of CMs in the cattle digestive system should be studied mainly to understand (i) safety concerns caused by the inclusion of clay particles into the digestive system and (ii) the possible role of CMs played in the rumen and later digestive pathway. In the first case, CMs such as bentonite, kaolinite, and montmorillonite are often used to adsorb toxins, improve feed efficiency, and stabilize gut pH; they can also pose potential health risks if not properly managed [127,128]. These interactions can alter the properties of CMs before they pass through to the feces, though little information is available about their fate once they are ingested. The release of ions, such as aluminum, calcium, magnesium, iron, and trace metals, results from the breakdown of clay structures under different pH conditions in the gastrointestinal tract of animals. Meanwhile, physical abrasion and microbial activities can lead to a reduction in particle size, enhancing the clay’s surface area and adsorption capacity, but also potentially altering its passage through the digestive system [19]. The report has shown that released aluminum from CMs found in the intestinal lumen and within the mucosal barrier, suggesting less absorption of zinc and other bivalent cations in the ruminal digestive system [129,130]. In addition, animal studies showed that CMs can cross the mucosa barrier, indicating potential aluminum-related neurotoxicity [131]. However, further investigation is needed to confirm this effect on ruminant animal models. Then, the presence of clay in the feces of ruminants provides insights into the clay’s journey and transformations throughout the digestive tract. Studying excreted clay helps in understanding nutrient and toxin adsorption, clay stability, and potential impacts on animal health [19,132]. The organo-mineral composition analysis on the feces of the studied animal may provide much information on the presence of clay particles in the feces sample.

In the case of modified clays, the fate analysis might be more complex, influenced by microbial adhesion, organic acids, biofilm formation, mineral weathering, organic matter stabilization, and the delivery of other active ingredients initially bound to the CMs [99,133]. For example, when feed is supplemented with iron-rich clay, the detection of iron in the feces of animals indicates several important factors related to the digestion, absorption, and metabolism of iron. Rumen microbes can produce organic acids during fermentation, which may enhance the solubility of iron from CMs [69]. Nevertheless, the final composition of iron in the feces will reflect the balance of iron that was dissolved, absorbed, and excreted. Feces may contain significant amounts of iron, both in free or available forms and as part of CMs, depending on dietary intake and microbial activity in the rumen. However, the quantification of the released iron in the feces is quite challenging because of the influences of microbes, diet, sample preparation, and techniques of determination. Although some advanced techniques, such as SEM/EDS, TEM/Cryo-TEM, X-ray diffraction, Fourier Transform Infrared Spectroscopy (FTIR), Thermogravimetric Analysis (TGA), Brunauer–Emmett–Teller (BET), and Inductively Coupled Plasma Mass Spectrometry (ICP-MS), have been applied for the characterization, quantification, and degradation of CMs, interpreting the results requires significant expertise due to the complexity of interactions between clay particles, dietary components, and ruminal fluid [134,135,136]. Moreover, to get the specific degradation of clays led by clay–microbe interactions, we must focus on other factors such as organic matter, pH, redox conditions, and competing ions. These factors can lead to multiple simultaneous processes such as sorption, precipitation, or reduction, making it difficult to isolate microbial-specific effects. Controlled laboratory experiments under varying conditions can help dissect these complex processes.

## 5. Conclusions and Outlook

**Figure 7:**
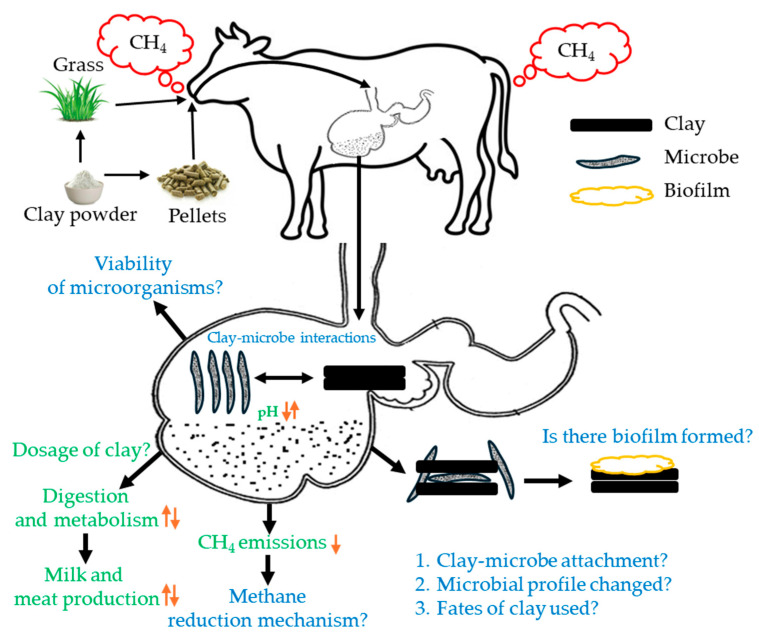
Key questions in understanding clay–microbial interactions for reducing CH_4_ emission from the rumen system. The solid black arrows are the process path, and the orange upward arrows indicate the increase, and orange downward arrows represent the decrease of the associated process.

Based on historical use and current research progress, we found that clay minerals can be used as a feed supplement to reduce cattle’s enteric methane generation. This can be achieved through their intervention with active microorganisms and biota, such as methanogenic archaea, bacteria, and protozoa. This can be done without harming their health or affecting livestock production. Clays may serve as carriers for methane-inhibiting additives, providing a safe, slow release that avoids stressing rumen microorganisms and gut physiology. Moreover, nutritional components resulting from clay dissolution may be added to the diet. However, depending on the clay dosage relative to diet matter, it may influence the passage rate of feed, thus affecting nutrient availability for the animal. Considering the complex interaction of clay minerals, feed, and the ruminal ecosystem, we have merged some findings in the scheme (Figure 7) and, at the same time, raised some questions that are critically important to explore the CH_4_ reduction mechanism.

However, to better understand clay–microbe interactions in reducing ruminal methane, more in vivo animal trials and vigorous sampling are needed to validate in vitro findings effectively. In addition, we must implement a cross-disciplinary research approach involving physicochemical, microbiological, and molecular experiments to address these hypothetical questions. By minimizing challenges in sample preparation and imaging, investigating clay–microbial interactions in the rumen could pave the way for developing effective mitigation strategies in future research (Figure 7). This approach may reveal critical insights into how clay can modulate microbial communities to reduce CH_4_ emissions, supporting more sustainable livestock practices.

## Figures and Tables

**Table 1:** Key physicochemical properties of major clay minerals, potentially used in feed additives [19,54,55,57].

Clay Types	Structure	Cation Exchange Capacity (CEC); [cmol(+)/kg]	Surface Area [m^2^/g]
Smectite (e.g., montmorillonite, saponite, nontronite), and illite-smectite. [Often found as bentonite in feed industry]	A 2:1 clay mineral with two tetrahedral sheets sandwiching an octahedral sheet	Exhibits a range of CEC (20–150) depending on specific mineral group. The range of CEC in illite-smectite clay is influenced by the ratio of illite to smectite layers	Comparatively high, depending on mineral types; smectite can be 40–800, while illite 10–100
Palygorskite/attapulgite; sepiolite	A 2:1 clay; fibrous morphology	3–20	40–180
Kaolin clay (e.g., kaolinite, halloysite)	A 1:1 clay mineral with one tetrahedral sheet directly bonded to one octahedral sheet	Kaolinite has a low CEC (5–15) due to its lower surface area and minimal isomorphous substitution, while halloysite’s CEC varies (5–40) depending on its hydration form (e.g., 7 Å or 10 Å)	About 5–40 for kaolinite, whereas it can be up to 190 for halloysite depending on its morphology and crystal damage

**Table 2:** Summary of recent studies on clay and clay minerals-based feed supplements added to the rumen.

Name of the Clay and Clay Minerals	Medium	Dosage	Possible Effects and Interactions	References
Iron-coated kaolinite (source clay *, West Lafayette, IN, USA)	Cell-line (Rumen Bacteria)	20 g/L	Microbial attachment, dissolution of Al, reduced iron, and reduced levels of CH_4_ were found	[69]
Bentonite	Rumen (in vitro)	-	Reduced ciliate protozoa that is partially responsible for methanogenesis	[105]
Bentonite	Rumen (in vivo)	50 g/cow/day	Increased levels of friendly microbes and reduced levels of pathogenic and endotoxin-producing bacteria have been found	[106]
Bentonite	Rumen (in vivo)	Bentonite: 4 to 8% of the total dry matter (DM), Saponite: 0.15 and 0.25 g	Improvement of microbial adhesion, cellulolysis, buffer capacity, metabolism, volatile fatty acids profile, ammonia, and reduced methanogenesis	[13,107]
Zeolite, bentonite, and sepiolite	Rumen (in vitro)	10 mg/g of total substrate	Improved ruminal fermentation and stabilized ruminal environment, better microbial balance, reduced CH_4_ emissions	[22]
Bentonite or montmorillonite	Rumen (in vivo)	20 g/kg DM	Reduced aflatoxin and increased digestibility, increased milk production, control NH_3_ levels, decreased CH_4_ production	[102]
Modified nano-montmorillonite	Rumen (in vitro)	0.05 (low) and 0.5 (high) g/kg DM	Reduced mycotoxin, Aflatoxin, NH_3_ levels, CH_4_ production, and promoted VFAs production	[14,61]
Illite	Rumen (in vitro and in vivo)	1% of the total DM	The population of total bacteria, protozoa, and methanogens were lower compared to the control	[108]

* source clay provided by Clay Minerals Society, USA.

## Data Availability

No new data were created or analyzed in this study.
